# Low Pre-Season Hamstring-to-Quadriceps Strength Ratio Identified in Players Who Further Sustained In-Season Hamstring Strain Injuries: A Retrospective Study from a Brazilian Serie A Team

**DOI:** 10.3390/sports11040089

**Published:** 2023-04-20

**Authors:** Filipe Veeck, Cassio V. Ruas, Matheus Daros Pinto, Rafael Grazioli, Gustavo Pacheco Cardoso, Thiago Albuquerque, Lucas Schipper, Henrique Gonçalves Valente, Victor H. Santos, Márcio Dornelles, Paulo Rabaldo, Clarice S. Rocha, Bruno Manfredini Baroni, Eduardo Lusa Cadore, Ronei Silveira Pinto

**Affiliations:** 1 Exercise Research Laboratory, School of Physical Education, Physiotherapy and Dance, Federal University of Rio Grande do Sul, Porto Alegre 90690-200, Brazil; rafael_grazioli@hotmail.com (R.G.); clarice.rocha@ufrgs.br (C.S.R.); edcadore@yahoo.com.br (E.L.C.); ronei.pinto@ufrgs.br (R.S.P.); 2 Brazilian Institute of Neuroscience and Neurotechnology, Institute of Physics Gleb Wataghin, University of Campinas, São Paulo 13083-854, Brazil; cassiovruas@gmail.com; 3 School of Medical and Health Sciences, Edith Cowan University, Joondalup 6027, Australia; m.pinto@ecu.edu.au; 4 Medical and Technical Department, Grêmio Foot-Ball Porto Alegrense, Porto Alegre 90250-590, Brazil; gugapacheco.fisio@gmail.com (G.P.C.); thiagopoa1@gmail.com (T.A.); schipper.fisioterapia@gmail.com (L.S.); nicovalente4@gmail.com (H.G.V.); victor.henrique408@gmail.com (V.H.S.); drmarciodornelles@hotmail.com (M.D.); rabaldo@live.com (P.R.); 5 Physical Therapy Department, Federal University of Health Sciences of Porto Alegre, Porto Alegre 90050-170, Brazil; bmbaroni@yahoo.com.br

**Keywords:** isokinetic, muscle strength balance, peak torque, hamstring strain injury, football

## Abstract

A common pre-season injury prevention assessment conducted by professional football clubs is the hamstring-to-quadriceps (H:Q) strength ratio calculated by peak torque (PT). However, it is debatable whether players that present low pre-season H:Q ratios are more susceptible to further sustaining in-season hamstring strain injuries (HSI). Based upon retrospective data from a Brazilian Serie A football squad, a particular season came to our attention as ten out of seventeen (~59%) professional male football players sustained HSI. Therefore, we examined the pre-season H:Q ratios of these players. H:Q conventional (CR) and functional (FR) ratios, and the respective knee extensor/flexor PT from the limbs of players further sustaining in-season HSI (injured players, IP) were compared to the proportional number of dominant/non-dominant limbs from uninjured players (UP) in the squad. FR and CR were ~18–22% lower (*p* < 0.01), whereas quadriceps concentric PT was ~25% greater for IP than UP (*p* = 0.002). Low scores of FR and CR were correlated (*p* < 0.01) with high levels of quadriceps concentric PT (r = −0.66 to −0.77). In conclusion, players who sustained in-season HSI had lower pre-season FR and CR compared to UP, which appears to be associated with higher levels of quadriceps concentric torque than hamstring concentric or eccentric torque.

## 1. Introduction

It is well documented that the occurrence HSI in a professional football club is an issue that can compromise individual and team success during a season, result in early career retirement, and result in financial hardships [1,2,3]. Despite significant effort made over the last years to identify risk factors and tests that can predict hamstring injuries, it remains debatable as to whether contemporary pre-season strength assessment methods can accurately inform coaches of those athletes that may be at greater risk of injury during the season.

Isokinetic strength assessment is generally used in football clubs’ medical departments during the pre-season [4,5], and one of the most commonly calculated variables is the hamstring-to-quadriceps (H:Q) strength ratios calculated by peak torque (PT) to potentially detect the risk of players further sustaining HSI during the season [6,7,8,9,10]. These ratios reflect the torque balance between hamstring and quadriceps, and an H:Q imbalance has been previously described as a potential cause of HSI incidence in sports that occur when the hamstring do not generate enough torque to counteract the anterior tibial translation and/or rotation during forceful knee extensions (e.g., acceleration, deceleration, change in direction) [6,7,11,12,13]. Traditionally, H:Q ratio is presented in two forms: (a) conventional ratio (CR), which is calculated by dividing the knee flexion concentric peak torque (PT) to the knee extension concentric PT and (b) functional ratio (FR), which is calculated by dividing the knee flexion eccentric PT by knee extension concentric PT. These torque measures are often obtained from isokinetic devices [14]. However, the assessment of H:Q ratios has recently received much scrutiny regarding its potential to inform or predict HSIs [15,16,17,18], even though large-cohort prospective trials have shown that low pre-season H:Q ratios were associated with a greater risk of sustaining an acute HSI [19,20]. Although controversy exists regarding this topic, these assessments have been a common practice for researchers, strength and conditioning coaches, and physical therapists to potentially screen for the risk of lower extremity injuries (e.g., HSI) in professional football players during pre-season [6,7,21].

Extensive focus to injury prevention-related research comes from European and Middle Eastern football leagues [15,16,22,23,24,25,26]. However, a large proportion of injuries also occur in South American football leagues [27,28]. For instance, a recent descriptive study performed in professional football teams from a southern state of Brazil showed that ~30% of players sustained an injury during a season, and that 38% of these injuries occurred in thigh muscles, with strain injuries being the most common (71.4%), especially in the hamstring (51.4%) [28]. Although it is difficult to compare injury rate data among studies due to the large differences that might exist in the sample size and number of seasons included, a previous study including players only from European professional football clubs showed that the prevalence of injuries in the hamstring was much lower than the one reported in the aforementioned study (~17% per season [22]). While differences in the number of football match fixtures and competitions per season exist, the potential risk factors leading to HSI in Brazilian Serie A football players have been poorly explored, which is surprising given the strength of this highly competitive league (i.e., Brazilian Serie A) [29], and that Brazil is one of the largest countries in the world where football is practiced at multiple levels.

In the 2018 season, we were very intrigued by the high number of HSI (~59%) sustained in a squad of a professional football team (i.e., a squad of thirty-eight players) playing the Brazilian Serie A league. Thus, given that this squad could offer a great possibility to investigate some of the potential causes of this high injury rate, we contacted the club to retrospectively analyze the isokinetic strength data from players that were recorded during that pre-season. Based upon the data provided by the football club concerning this particular season, the purpose of this study was to examine whether the traditional H:Q ratios calculated by PT (assessed during the pre-season) of players that further sustained in-season HSI (i.e., injured players—IP) was different than players that did not sustain any injury (i.e., uninjured players—UP). A secondary aim was to explore whether individual quadriceps and hamstring PT for each muscle action differed between those groups of players and were associated to the H:Q ratio scores. Given the experimental design and exploratory nature of the present study, no initial hypothesis was raised. However, due to the common use of H:Q strength ratios for general muscle strength imbalance assessment, knee joint stability monitoring, description of functionality and strength properties, and as potential tool for injury prevention and rehabilitation [7,11,12,13], it is expected that the results from this study can give insights into the most appropriate interpretation and offer practical recommendations based on this assessment during pre-season of Brazilian Serie A professional football players.

## 2. Materials and Methods

### 2.1. Overview

One professional football club (i.e., including the players from the squad) from the Brazilian Serie A league agreed to provide the data related to the pre-season isokinetic assessment (quadriceps and hamstring PT, CR, and FR) and the prevalence of HSI in the squad during the 2018 season. This club participated of the most relevant national (i.e., Brazilian Cup, and Brazilian Serie A League) and international level leagues (e.g., Libertadores da America Cup and FIFA Club World Cup). The Brazilian Serie A league is one of the most valuable leagues on the American continent. In fact, the International Federation of Football History and Statistics (IFFHS) has recently ranked the Brazilian Serie A as one of the men’s strongest football leagues in the world (ranking launched since 1991) [29]. The players completed a total 4–5 football training sessions (~90 min per session) and 1–2 matches per week during January to December—a total of 73 matches and 6570 min of match in the season. Training took place at the club’s own training center, which is considered as one of the most structured in Latin America. It includes two fields with official measurements and two smaller grass fields for specific training/small-sided games and/or goalkeeping practices. It also has many fitness gyms, health departments (e.g., physiotherapy, treatment, care, recovery, physiology) and other recovery and training facilities (indoor and outdoor swimming pools of different sizes and rooms with temperature regulation among others) for the use of the players.

Based upon the data provided from the football club, players only completed one resistance training session a week given the season’s highly congested fixture calendar. The isokinetic assessments were performed during pre-season by an experienced evaluator (early January). All injury incidents were recorded throughout that season, being confirmed through clinical examinations by the medical and physiotherapy staff with the use of ultrasonography and/or magnetic resonance imaging. Ethical approval for this retrospective investigation was obtained from the Universidade Federal do Rio Grande do Sul, Brazil (Project n° 2.903.811).

### 2.2. Participants

The squad originally consisted of thirty-eight male professional football players. However, to reduce the number of external factors influencing our analyses, the goalkeepers (n = 4) were excluded from analysis, and only players that had not sustained recent HSI (i.e., in the last 6 months) and/or played more than 8 matches (300 match/minutes)—~11% of total matches and ~5% of match/min, respectively—during the season were included in the sample. This resulted in a total of 17 players included in the analyses of the present study. During the season, 10 out of those 17 (~59%) players in the squad sustained HSI. The mean and standard deviation (SD) of the sample’s characteristics, playing position, number and time of matches played during the season, injury grade classification, involvement of the injured leg, and days of recovery until return to play are summarized in Table 1.

### 2.3. Injury Definition, Diagnosis, and Classification

The HSI were defined as an acute pain in the posterior thigh that occurred during training or matches, resulting in the immediate termination of play and inability to participate in the following commitment to the squad (i.e., a training session or match) [30]. The HSI grades were classified as follows:

- Grade 1: Minimal muscle elongations identified (i.e., corresponding to less than 5% of the muscle volume or cross-sectional diameter).
- Grade 2: Partial muscle ruptures identified (i.e., corresponding from 5% to 50% of the muscle volume or cross-sectional diameter).
- Grade 3: Muscle tears with complete retraction. Usually, these lesions are clinically evident because the muscle belly forms a real mass, and a gap can be palpated between the retracted ends of the muscle [31].

At the conclusion of the season, all data were stored in a central database with access given to physicians and physical therapists of the club. These were further provided by the club at our request for a retrospective analysis.

### 2.4. Isokinetic Protocols

Maximal knee extensor, flexor concentric, and knee flexor eccentric PT were measured using a Biodex isokinetic dynamometer (Biodex, System 4 Pro™, Byodex Medical Systems, New York, NY, USA). All tests were conducted on an isokinetic dynamometer located in the club, and the test procedures followed the same settings of previous studies [6,7,8]. For this, the athletes sat on the chair of the machine with their hips positioned at 85° of the dynamometer and had straps placed across their thighs, hips, and chest to minimize extraneous movement. The lateral epicondyle of the tested knee was aligned with the dynamometer’s axis of rotation, and the machine’s lever arm was attached to the shank at 2 cm above the lateral malleolus of the ankle. Both the dominant (defined as preferred leg when kicking a ball) and the non-dominant knees were assessed through a 90° range of motion (from 90° of knee flexion to 0° of full extension).

Prior to testing, participants first performed a 10 min warmup at a comfortable pace on a cycle ergometer (Life Fitness, 95 Ci Upright Bike, São Paulo, SP, Brazil), followed by a specific isokinetic warmup of 10 submaximal concentric knee extension-flexion contractions at 120°·s^−1^. During this specific warmup, they were instructed to perform contractions at self-selected submaximal joint moments (perceived effort) in the first three repetitions and then gradually increase their torque production in the last two repetitions [8]. Then, after a resting period (~90 s), the participants performed a total of five maximal concentric knee extension-flexion muscle contractions at 60°·s^−1^ [6], which measured their knee extension (quadriceps) and knee flexion (hamstring) maximal concentric torques. Finally, athletes were tested over five maximal eccentric knee flexion muscle contractions at 60°·s^−1^ [6], which measured their hamstring maximal eccentric torque. A resting period of 120 s was provided between the concentric and eccentric trials, and the athletes were instructed to “push and pull as hard and as fast as possible” during every repetition [32].

It is important to note that participants were already familiar with the testing procedures, as they were often tested as part of their screening routine before the start of every season. Torque signals were recorded after gravity correction and sampled at 100 Hz using the dynamometer’s software (Biodex, System 4 Pro™, Byodex Medical Systems, New York, NY, USA). The highest PTs of each muscle action across all repetitions and trials were used for further analysis.

### 2.5. Muscle Strength Imbalance Measurements

The H:Q ratios (i.e., CR and FR) were calculated by considering the highest PTs of each muscle action recorded at 60°·s^−1^. The CR was calculated by dividing knee flexion concentric PT by knee extension concentric PT [13]. The FR was calculated by dividing knee flexion eccentric PT by knee extension concentric PT [13].

### 2.6. Statistical Analysis

Data normality was verified using the Shapiro–Wilk test. The dependent variables measured during pre-season (CR, FR, quadriceps and hamstring concentric PT, and hamstring eccentric PT) were compared between IP (n = 10) and UP (n = 7) limbs by independent t-tests. A Bonferroni correction was used to reduce the likelihood of type I error due to the multiple t-test comparisons. Since the IP had HSI diagnosed in four dominant (33%) and six non-dominant (66%) limbs, statistical analyses between groups were confounded by limb dominance and sample size inequality. To overcome this issue, an approximately proportional number of dominant (33%) and non-dominant (66%) limbs of the UP was used to allow for an equivalent between-group comparison of the raw data. Therefore, two dominant and five non-dominant limbs of the UP group were randomly selected using the random function in the Excel software (Microsoft Excel, version 16.26). Moreover, Pearson product-moment (r) correlation tests were used to examine the relationships between H:Q ratio (CR and FR) scores and the levels of quadriceps and hamstring PT for each muscle action in all players. The level of significance (α) was set at 0.05, and all statistical procedures were performed using the Statistical Package for Social Science (SPSS) version 20.0 (IBM SPSS Inc., Version 28.0.1.0, Chicago, IL, USA). The results are reported in means ± standard deviation (SD).

## 3. Results

**Figure 1:**
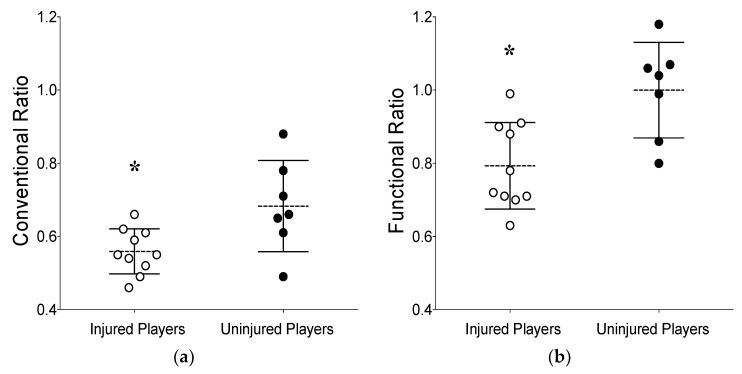
Conventional (CR) (**a**) and functional (FR) (**b**) hamstring-to-quadriceps ratios between limbs of injured (n = 10) and uninjured (n = 7) football players. Data are presented as means ± SD, and circles represent individual players from each group. * Indicates significant difference from uninjured players (*p* < 0.05).

Significant differences between IP and UP were found for CR, FR, and quadriceps PT. CR was lower for IP (0.56 ± 0.06; range: 0.46 to 0.66) than UP (0.68 ± 0.12; range: 0.49 to 0.88) [t(15) = −3.18, *p* = 0.006; Figure 1a]. Similarly, FR was lower for IP (0.79 ± 0.12; range: 0.63 to 0.99) than UP (1.00 ± 0.13; range: 0.86 to 1.07) [t(15) = −3.72 *p* = 0.002, Figure 1b]. However, quadriceps PT was greater for IP (207.8 ± 22.6 N·m; range: 166.0 to 235.1 N·m) than UP (156.9 ± 40.8 N·m; range: 87.7 to 207.9 N·m) [t(15) = 3.34, *p* = 0.004]. No significant differences between groups were found for hamstring concentric [t(15) = 1.22, *p* = 0.24] and eccentric PT [t(15) = 0.65, *p* = 0.52] (Table 2).

**Figure 2:**
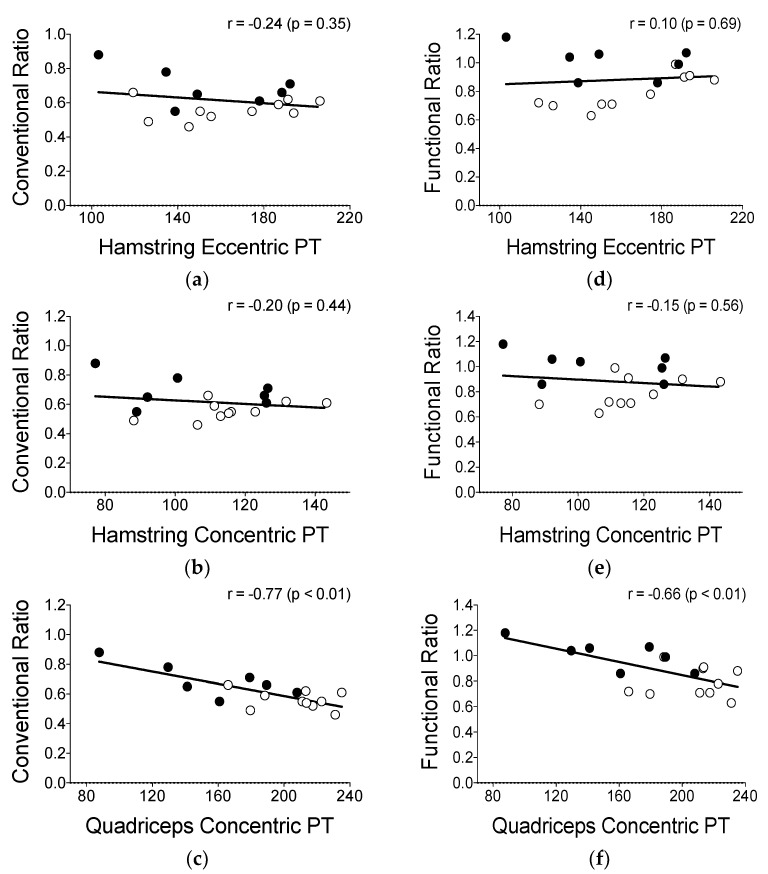
Correlations between conventional and functional ratios in relation to hamstring eccentric peak torque (PT), hamstring concentric PT, and quadriceps concentric PT of football players. Data of limbs of uninjured players are shown in black circles (n = 7), and data of limbs of injured players are shown in white circles (n = 10).

Low scores of CR and FR were significantly correlated with high levels of quadriceps concentric PT (r = −0.77 to −0.66, all *p* < 0.01). Visual inspection to the individual data plots revealed that those associations were more pronounced for the IP than UP (Figure 2c,f). However, no significant correlations (*p* > 0.05) were evident for CR or FR and hamstring concentric and eccentric PT (Figure 2a,b,d,e).

## 4. Discussion

In the present study, we retrospectively examined whether the pre-season H:Q strength ratios and knee extensor/flexor PT were different between injured and uninjured players (i.e., IP and UP, respectively) in a professional football club from the Brazilian Serie A league that presented an unusual and very high rate of HSI during one particular season. The results showed that that pre-season H:Q ratios (conventional and functional ratios; CR and FR) were lower, and concentric PT was higher for IP than UP. Furthermore, low scores of FR and CR were correlated with high levels of quadriceps concentric PT (r = −0.66 to −0.77), and an analysis of individual data indicated this association was more evident in the IP than UP. Therefore, players that sustained in-season HSI showed lower pre-season FR and CR and greater quadriceps concentric PT prior to sustaining an injury compared to UP. These findings suggest that players exhibiting higher quadriceps concentric torque levels without equivalent hamstring torque during pre-season assessment (i.e., leading to an increased H:Q strength imbalance) could be at a greater risk of sustaining in-season HSI.

Assessments involving traditional H:Q ratios or hamstring eccentric PT have been often included during pre-season routines of professional football clubs to monitor the potential risk of players sustaining in-season HSI [8,10,33]. The dissemination of the H:Q strength ratio assessment comes from early research showing that lower extremity injuries, such as HSI and anterior cruciate ligament (ACL), are likely to occur when the hamstring are not capable of generating equivalent torque to decelerate the high levels of quadriceps torque needed during knee extension anterior tibial shear or rotation movements [6,12,13,19]. The classic study from Croisier et al. [19] showed that professional football players with low pre-season imbalances (including H:Q ratios) were four to five times more likely to sustain HSI. In line with this, our findings revealed that the 59% of players in the squad that sustained in-season HSI also presented lower pre-season FR and CR compared to their uninjured counterparts.

It is generally accepted that low levels of hamstring PT are the main contributors to lower H:Q ratio levels and thus a potential increased HSI risk [13,19,34,35]. However, in the present cohort, low H:Q ratios in the IP were mainly mediated by high levels of quadriceps concentric PT. A closer look at the individual data also revealed that injured individuals had overall greater levels of quadriceps PT (>160 N·m), thus resulting in low CR (<0.7) and FR (<1.0) scores (Figure 2c,f). Interestingly, the IP group had approximately similar levels of hamstring eccentric strength to UP. This may support the idea shown by a recent study, which demonstrated that pre-season hamstring eccentric strength by itself was not associated with a greater risk of in-season HSI in 326 collegiate athletes (including football players), after controlling for known risk factors (e.g., age, prior HSI, and sex differences) [36]. Taken together, these findings seem to support that special care should be taken during pre-season to monitor whether the quadriceps concentric torque is unproportionally higher than the hamstring, which could in turn also lead to potentially greater knee joint instability and a consequent increase in the risk of HSI incidence in those players. If that is the case, targeted strength and conditioning programs that aim for equivalent strength and muscle activation patterns between both hamstring and quadriceps are necessary. However, in circumstances in which football players present the strength of the hamstring muscle group lower than that of the quadriceps, it may still be important to specifically strengthen the hamstring using isokinetic or traditional resistance training exercises to correct for such an imbalance.

We acknowledge that this study had some limitations. First, we limited our retrospective analysis to only one professional football team from the Brazilian Serie A because of the opportunity we had to investigate potential factors that can result in injury by examining data from a squad that presented a very high HSI rate during a particular season. However, it would be interesting to examine whether similar differences in the pre-season H:Q ratios between IP and UP groups are present in larger samples of South American professional football squads, leagues, and players in future studies. Second, the data provided to us by the football club only included isokinetic assessments taken during pre-season, but other in-season assessments of the players were not available. It would be interesting to further explore data from more time points over the season to further explore H:Q ratio differences and injury incidence from the players in the squad. Similarly, it was not possible to gather further information regarding the duration of the phases and the validity of the training protocol that were used by the players during that specific season. We consider this important data that should be recorded by further studies and related to H:Q ratio and injury rate data of a football squad. Finally, the data we had access to for this study only included isokinetic PT assessments, but it would be important to also explore other neuromuscular measurements in professional football players to further understand additional mechanisms that can influence the H:Q strength balance (i.e., torque produced at multiple angles of range of motion, explosive strength, muscle size, muscle fatigue, muscle activation) [11] and how they relate to an in-season HSI incidence. It is also important to emphasize the exploratory character of the present study; thus, no causal relationship was determined.

## 5. Conclusions

The present study revealed that, in a professional South American football squad that presented a very high rate of HSI during one season, the players who sustained HSI during the course of the season were the ones that previously showed lower pre-season H:Q FR and CR. The low H:Q ratios of these players were particularly associated with high levels of quadriceps concentric PT without equivalent hamstring eccentric PT. Therefore, although debate exists regarding the use of strength imbalance assessments as screening tools to prevent or predict HSI (i.e., also given the multifactorial nature of such injuries), our results suggest that H:Q ratios could still be important variables to be monitored in professional football players during the pre-season; at least in the highly injured squad that we examined, data seems to support the contention that professional players that have greater muscle strength imbalances during the pre-season might be the ones who potentially sustain in-season HSI. Furthermore, the results from the present study enhance the understanding of the specific muscle actions that can be mostly associated with H:Q strength ratio imbalances. This information may also be used to inform best training, injury prevention, and/or rehabilitation practices for physical therapists, strength and conditioning coaches, and sport scientists from the Brazilian Serie A and/or other important competitive professional leagues. For instance, based upon our findings, it may be that resistance training programs that allow an equivalent hamstring-to-quadriceps strength balance and proportional muscle activation patterns for dynamic knee joint stability could be potentially effective to reduce the occurrence of in-season HSI in the squad. Nevertheless, the relatively small sample of the present study, including data from a single professional football squad, does not allow a complete generalization of our findings to other teams/players. Follow-up studies are necessary to further investigate and inform best resistance training approaches, which may depend on the characteristics of the squad and the pre-season isokinetic hamstring- and quadriceps-specific strength data leading to strength imbalances in football players.

## Figures and Tables

**Table 1:** Participants’ characteristics.

	Injured Players (IP) (n = 10)	Uninjured Players (UP) (n = 7)
Age, y	27.9 ± 4.9	26.4 ± 5.2
Body mass, kg	80.2 ± 8.4	77.3 ± 7.8
Height, cm	179.9 ± 4.3	108.9 ± 4.8
Player position, n	Defenders: 4 Midfielders: 2 Attackers: 4	Defenders: 3 Midfielders: 3 Attackers: 1
Total number of games played during the season, n	33.5 ± 13.0	37.7 ± 19.5
Total time of games played during the season, min	2199.3 ± 1000.1	3049.0 ± 1784.3
Games until HSI, n	18.3 ± 12.1	NA
Time of game until HSI, min	1175.8 ± 1025.6	NA
HSI grade, n	Grade 1: 2 Grade 2: 7 Grade 3: 1	NA
Limb dominance of HSI, n	Dominant: 4 Non-dominant: 6	NA
Days off due to HSI, n	28.1 ± 24.7	NA

The results are reported in mean ± SD. HSI, hamstring strain injury; NA, not applicable.

**Table 2:** Quadriceps concentric and hamstring concentric and eccentric peak torque (PT) between limbs of injured (n = 10) and uninjured (n = 7) football players.

Isokinetic Strength Measures	Injured Players (IP) (n = 10) [95% CI]	Uninjured Players (UP) (n = 7) [95% CI]	*p* Value
Quadriceps concentric PT (Nm)	207.8 ± 22.6 * [166.0 to 235.1]	156.9 ± 40.8 [87.7 to 207.9]	0.004
Hamstring concentric PT (Nm)	115.7 ± 14.8 [88.2 to 143.3]	104.1 ± 21.9 [77.2 to 126.5]	0.241
Hamstring eccentric PT (Nm)	164.9 ± 29.9 [119.3 to 206.1]	153.8 ± 33.5 [103.2 to 192.2]	0.522

Data are presented as means ± SD. * Indicates significant difference from uninjured players (*p* < 0.05).

## Data Availability

Data are available on request to the corresponding author.
